# Multivariate analysis of factors for failed continuous bladder irrigation in hemorrhagic cystitis patients after hematopoietic stem cell transplantation

**DOI:** 10.1186/s12894-020-00757-5

**Published:** 2020-11-10

**Authors:** Wenbo Yang, Yiqing Du, Zhan Qu, Wenjun Bai, Luping Yu, Xiaopeng Zhang, Qi Wang, Xiaowei Zhang, Qing Li, Tao Xu

**Affiliations:** Department of Urology, Peking University People’s Hospital, 11 South Xizhimen Street, Beijing, 100044 China

**Keywords:** Continuous saline bladder irrigation, Transplant-related adverse events, Hemorrhagic cystitis, C-reactive protein, Hematopoietic stem cell transplantation, CRP

## Abstract

**Background:**

Continuous bladder irrigation (CBI) and proper adjustment of saline irrigation speed are important to avoid CBI failure in hemorrhagic cystitis (HC) patients after allogeneic hematopoietic stem cell transplantation (HSCT). Nevertheless, too fast irrigation speed could take away the patient's much heat, contribute to blood coagulopathy, and increase the nursing workload. Evaluation of risk for CBI failure remains an unmet clinical need.

**Methods:**

The general information, clinical characteristics, and consultation records of HC patients in 1380 patients with hematopoietic stem cell transplantation in our center from 2017 to 2019 were analyzed retrospectively. The receiver operating characteristic (ROC) curve was used to calculate the cutoff point of the continuous variable, and multivariate logistic regression was used to analyze the risk factors affecting CBI failure in HC patients.

**Results:**

The incidence of HC after HSCT was 23%. A total of 227 patients with HC above grade 2 were included. Univariate analysis showed that CRP, age, platelet counts, onset time after transplantation, albumin, and hemoglobin were associated with CBI failure in the short-term (*P* < 0.05). ROC curve and multivariate logistic regression analysis showed that CRP > 8.89 ng/ml (RR = 7.828, 95% CI 2.885–21.244), age < 14.5 years (RR = 9.940, 95% CI 3.219–30.697), and onset time of HC > 37d after transplantation (RR = 7.021, 95% CI 2.204–22.364), were independent risk factors for failure of CBI (*P* < 0.05).

**Conclusions:**

The study identified CRP > 8.89 ng/ml, age < 14.5 years, and onset time of HC after HSCT > 37d are independent factors for failure of CBI, which could be combined to allow stratification of HC after HSCT patients into low-, intermediate- and high-risk subgroups of CBI failure.

## Background

Hemorrhagic cystitis (HC) after allogeneic hematopoietic stem cell transplantation (HSCT) is characterized by diffuse inflammation and hemorrhage of the bladder mucosa. Its clinical manifestation, severity, and prognosis vary greatly. It has been reported that the incidence of HC, as one of the major complications in allogeneic HSCT, is 14–30% [1–4]. Refer to the Droller’s HC classification (Table 1), grade I means only microscopic haematuria, and gross hematuria means grade II or higher. Conservative observation, hydration, alkalization of urine, diuretics, and antiviral therapy were efficient for most HC patients with grade I or II, while continuous bladder irrigation (CBI) was required for some grade II, III, and IV patients to avoid urinary tract obstruction caused by blood clots in the bladder.

**Table 1:** Hemorrhagic cystitis grade

Grade I	Microscopic hematuria
Grade II	Macroscopic hematuria
Grade III	Macroscopic hematuria with small clots
Grade IV	Gross hematuria with massive clotting, causing urinary tract obstruction, requiring instrumentation for clot evacuation

Patients with this allogeneic HSCT have abnormal immunity, coagulopathy [5], and graft-versus-host disease (GVHD). For urinary tract obstruction of HSCT patients, surgical treatment is associated with mortality and effects were minimal. CBI and proper adjustment of saline irrigation speed are important to avoid CBI failure. Bladder irrigation pressure and speed were dependent on the severity of hematuresis, risk of clots and patient’s comfort. As the irrigation continues, the urine should become pink and clear, especially in severe hematuresis [6]. However, we should maintain patient’s comfort at the same time. Nevertheless, too fast irrigation speed could take away the patient's much heat, contribute to blood coagulopathy, and increase the nursing workload.

Doctors often believe that the grading of HC is the most reliable factor to predict the failure of CBI for HC, which is not practical in clinical practice. Many patients were diagnosed with grade IV of HC as bladder irrigation failed. It means HC grade classification of the moment is not of enough practical significance in guiding the saline irrigation speed for HC to prevent CBI failure. Therefore, it is an unsolved clinical need to predict reliably the CBI failure of HC. We hypothesized that patients with HC grade II or higher had some clinical characteristics to predict the risk of CBI failure. We report an analysis of HC incidence and treatment outcome in our center.

## Methods

The study was approved by the institutional review board in our center. The general information, clinical characteristics, and consultation records of HC patients in 1380 patients with HSCT in our center from 2017 to 2019 were analyzed retrospectively. According to the consultation records in our center, if the irrigation line was completely blocked in CBI, it is determined as CBI failure of the patient in our center from 2017 to 2019. The following clinical parameters were collected: general information, the primary diseases, onset time of HC after HSCT, haploidentical HSCT, sex-mismatch in recipients, cytomegalovirus (CMV) viremia, EB viremia, hemoglobin, platelets, serum creatinine, C-reactive protein (CRP), and serum albumin in the presence of gross hematuria. If gross hematuria or clots are persistent, CBI is indicated in our institute from 2017 to 2019. All the patients with HC above grade 2 were treated with hydration, alkalization, diuretic equally. When viremia was found, ganciclovir or sodium phosphonate was used for antiviral treatment and human immunoglobulin to enhance immunity treatment until the viremia turned negative.

SPSS 26.0 statistical software was used for data analysis and data processing. The receiver operating characteristic (ROC) curve was used in measurement data to determine the appropriate cut-off point of predicting CBI failure, by which measurement variables were divided into binary variables. Univariate analysis and multivariate logistic regression were used to analyze the risk factors affecting CBI failure in HC patients. Comparisons of the rate of CBI failure across groups were made using analysis of chi-squared tests for categorical and binary variables. The difference was statistically significant at 0.05.

## Results

Among the 1380 patients with allogeneic HSCT in our center, a total of 311 (22.5%) patients were diagnosed with HC. HC was grade I, II, III, and IV in 84 (27.0%), 107 (34.4%), 73 (23.5%), and 47 patients (15.1%), respectively. Among these patients, 227 patients with HC of degree 2 or higher were included. Table 2 shows the demographic and clinical characteristics of these patients. The patients' primary diseases included acute myeloid leukemia (53, 23.3%), myelodysplastic syndrome (25, 11.0%), acute lymphoblastic leukemia (91, 40.1%), acute mixed cell leukemia (6, 2.6%), Hodgkin's lymphoma (3, 1.3%), aplastic anemia (32, 14.1%), T-cell lymphoma (1, 0.4%), and others (16, 7.0%).

**Table 2:** Patients of grade II and above characteristics

Number of patients	227
Age (mean ± SD), years	27.0 ± 14.5
Women, n (%)	104 (45.8%)
Grade, n (%)	
II	107 (47.1%)
III	73 (32.2%)
IV	47 (20.7%)
Primary diseases, n (%)	
Acute myeloid leukemia	53 (23.3%)
Myelodysplastic syndrome	25 (11.0%)
Acute lymphoblastic leukemia	91 (40.1%)
Acute mixed cell leukemia	6 (2.6%)
Hodgkin's lymphoma	3 (1.3%)
Aplastic anemia	32 (14.1%)
T-cell lymphoma	1 (0.4%)
Others	16 (7.0%)
CMV virus positivity, n (%)	84(37.0%)
EB virus positivity, n (%)	35(16.3%)
Cyclophosphamide exposure, n (%)	201(88.5%)
CBI failure	39(17.2%)

A total of 93 in 311 (29.9%) patients with severe hematuria were undergone CBI. Among 311 HC patients, a total of 39 patients (12.5%) failed in CBI. Among them, 19 cases underwent cystoscopy to remove blood clots and urinary tract obstruction after failed manual flushing by experienced urologists, and 3 cases were transferred to the pediatric centers for cystoscopy due to their age.

### Establishment of the cut-off value of the continuous variable

**Fig. 1:**
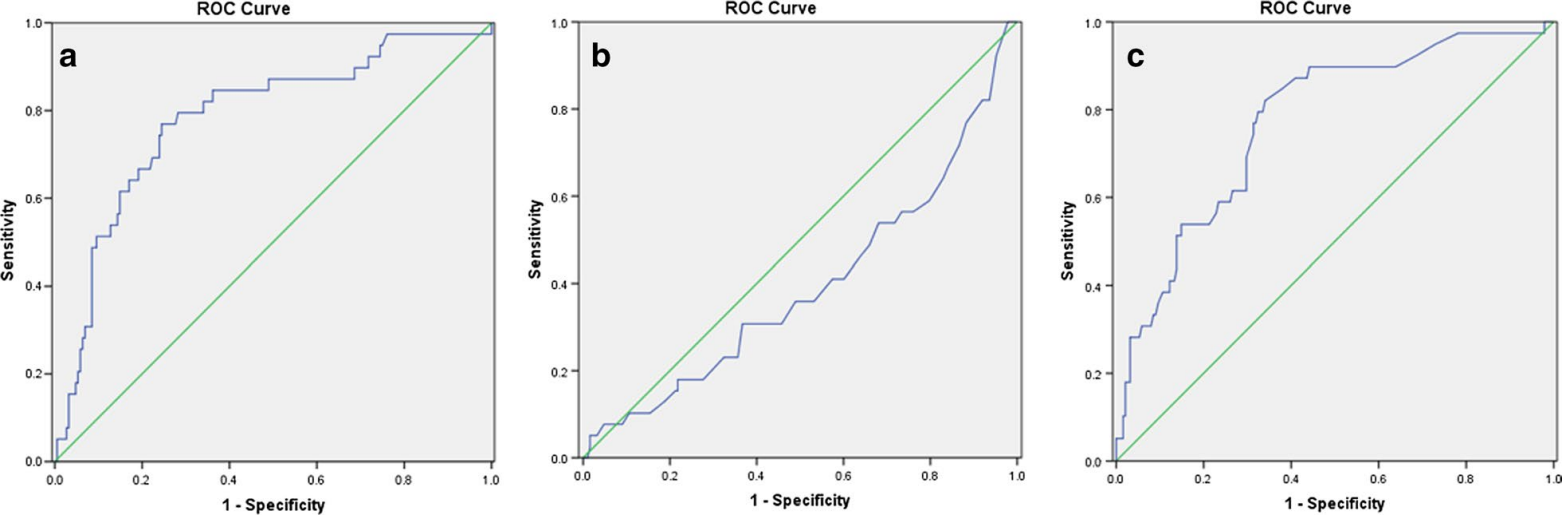
ROC curve to predict CBI failure. The values plotted on the curves indicate specificity and sensitivity at the points that are closest to the upper-left corner. **a** CRP, **b** age, **c** onset time of HC

To determine the cutoff points of CRP, age, platelets count, post-transplantation onset time, serum albumin, and hemoglobin in the entire cohort, the ROC curve for CBI failure was plotted (Fig. 1). The area under the curve (AUC) of CRP was 0.784. The sensitivity and the specificity were 0.769 and 0.755 respectively as a CRP of 8.89 mg/dL was defined as the cutoff point. As for the onset time of HC after HSCT, the AUC was 0.773 and the cutoff value was determined as 37d by ROC curve, with a specificity of 0.660 and a sensitivity of 0.821. The cut-off values of age, platelet count, albumin, and hemoglobin were 14.5 years, 29.5 × 10^9^/L, 36.05 g/L, and 83.5 g/L, respectively. Then the impact of CRP and other continuous variables were recoded into binary variables and evaluated in the univariate analysis according to the cutoff point.

### Univariate analysis of CBI failure (Table 3)

**Table 3:** Results of uni- and multivariate analyses

Variable	Univariate analyses			Multivariate analyses		
	RR	*P*	95% CI	RR	*P*	95% CI
Age < 14.5 years	2.746	0.005	1.322–5.702	9.940	0.000	3.219–30.697
Male gender	0.767	0.451	0.385–1.531			
Haploidentical HSCT	0.728	0.683	0.158–3.362			
Gender difference	0.965	0.926	0.454–2.052			
Onset time of HC > 37d	8.857	0.000	3.704–21.177	7.021	0.001	2.204–22.364
platelet count < 29.5*10^9^/L	2.724	0.005	1.335–5.558	1.892	0.186	0.736–4.862
Albumin < 36.05 g/L	3.943	0.004	1.473–10.553	2.377	0.145	0.741–7.623
CRP > 8.89 mg/dL	10.290	0.000	4.551–23.264	7.828	0.000	2.885–21.244
hemoglobin < 83.5 g/L	3.010	0.002	1.468–6.172	1.537	0.378	0.591–4.001
CMV positivity	1.078	0.836	0.530–2.192			
EB positivity	1.415	0.434	0.591–3.385			

Gender difference: gender difference between donor and recipient

In this study, among the 123 male patients, 19 (8.4%) were failed in CBI, compared with 20 among 104 female patients (8.8%). There was no correlation between gender and CBI failure (*P* = 0.451). The age in the whole cohort was 27.0 ± 14.5 (IQR 15–36) years. Among the 54 patients with age < 14.5 years, 16 (7.0%) failed in CBI, and among the 173 patients with age ≥ 14.5 years, 23 (70.4%) failed in CBI. Age < 14.5 years was significantly associated with CBI failure (RR = 2.746, 95% CI 1.322–5.702, *P* = 0.005).

In 227 patients, the onset time of HC after HSCT was right skewness, and the median and mean for the whole cohort was available for 33.0 d and 59.1 d (IQR 24.0 d-60.0 d). Among the 96 patients with an onset time of HC after HSCT more than 37 d, 32 (33.3%) underwent CBI failure. On the contrary, only 7 (5.3%) among the 131 patients with an onset time of HC after HSCT fewer than 37d failed in CBI. Onset time of HC after HSCT more than 37d was significantly associated with CBI failure in univariate analysis (RR = 8.857, 95% CI 3.704–21.177, *P* < 0.001).

In the study, the mean and median of CRP in the whole cohort were available for 12.08 mg/dL and 4.08 mg/dL (IQR 1.21–11.84). Among the 151 patients with CRP ≤ 8.89 mg/dL, 9 (5.96%) underwent CBI failure. Among the 76 patients with CRP > 8.89 mg/dL, 30 (39.47%) underwent CBI failure. CRP > 8.89 mg/dL was significantly associated with CBI failure (RR = 10.290, 95% CI 4.551–23.264, *P* < 0.005). In the univariate analysis palates count < 29.5 × 10^9^/L (RR = 2.724, 95% CI 1.335–5.558, *P* = 0.005), albumin < 36.05 g/L (RR = 3.943, 95% CI 1.473–10.553, *P* = 0.004), and hemoglobin > 83.5 g/L (RR = 3.010, 95% CI 1.468–6.172, *P* = 0.002) were also significantly associated with CBI failure.

Nevertheless, in the univariate analysis gender difference between donor and recipient, primary disease type, haploidentical HSCT, CMV positivity, and EB positivity (*P* > 0.05).

### Multivariate analysis of CBI failure (Table 3)

In multivariate analysis, CRP > 8.89 ng/ml (RR = 7.828, 95% CI 2.885–21.244, *P* < 0.001), age < 14.5 years (RR = 9.940, 95% CI 3.219–30.697, *P* < 0.001), and onset time of HC > 37d after transplantation (RR = 7.021, 95% CI 2.204–22.364, *P* = 0.001) were independent risk factors for failure of CBI.

### Risk groups of CBI failure

Each patient was then assigned to one of three risk groups: those with zero or one risk factors (favorable risk), those with two risk factors (intermediate risk), and those with three risk factors (poor risk). Of the 160 (42%) patients deemed to be in the favorable-risk group, only 8 (5%) failed in CBI. In contrast, 88.9% of the poor-risk group, failed in CBI. There was a significant difference in the rate of CBI of the three risk groups (*P* < 0.0001).

## Discussion

Urologists are often consulted by hematologists about the failure of CBI in HC patients, which may require manual processing of blood clots and even surgical intervention in patients with immunity deficiency and hematopoiesis. In grade II, III, and all IV of HC patients related to HSCT, CBI is suggested to prevent large blood clotting in bladder forming and lower urinary tract obstruction to allow adequate catheter drainage. Although adjusting the speed of bladder saline irrigation dependent on the color of the drainage from the catheter can avoid the failure of CBI to some extent [7], medical nursing providers always slow down the speed of irrigation in any grades of HC patients for various reasons limiting CBI prophylactic value, such as hypothermia [4, 8]. Notably, if CBI failure was not resolved in time and CBI was not suspended, iatrogenic rupture of the bladder or renal function damage could be caused. Evaluation of risk for CBI failure remains an unmet clinical need. Compared with radiation cystitis, although the pathological changes and symptoms of HC related to HSCT are mainly in the bladder, its diagnosis and treatment can not bypass the special immune deficiency and abnormal hematopoietic function of HSCT, which means higher significant morbidity and mortality in surgical treatment. Through a large sample retrospective study in our center, the data of 1380 patients who received HSCT were analyzed retrospectively. The incidence of HC after HSCT was 23% in our center. The CRP, platelet count, and hemoglobin of patients with gross hematuria were analyzed statistically, and the independent risk factors of CBI failure were discussed. In the study, CRP was firstly found to be an independent risk factor for CBI failure, and its cutoff point CRP > 8.89 mg/dl was determined. Up to the date, CRP has not been reported as independent risk factors for HC grade or treatment outcome. In the current study, we have confirmed that patients with the onset time > 37d of HC after transplantation are more likely to fail in CBI. This is consistent with previous studies. Johnston et al. found that delayed HC onset time was an independent risk factor for CBI of HC in children [1]. Our study further determined the cutoff point and confirmed and that HC onset time > 37d is still an independent risk factor for CBI failure.

Within 3 days after the pretreatment of allogeneic HSCT, HC was mainly contributed to the toxicity of cyclophosphamide (CY) in the pretreatment. When acrolein, the metabolite of CY, combines with the epithelial tissue of bladder mucosa, it will cause extensive damage to the mucosa of the patients. At the same time, acrolein is more likely to form crystals and deposits in the kidney or bladder in the acid environment, which aggravates the bleeding and necrosis of the mucosa. Nevertheless, previous studies showed that the HC after 3 days of HSCT was mainly related to the infection of CMV, adenovirus, BKV [4], JC virus [9], and so on. Stanchi [10] et al. reviewed adverse events of 214 recipients undergoing HSCT in children, and the results indicated that CRP increased significantly during sepsis / SIRS, bacteremia graft rejection, liver or intestinal GVHD, and viremia. In this study, CRP in hematuria was found to be an independent risk factor for CBI failure in HC after HSCT, and CRP > 8.89 mg/dl of gross hematuria stage is superior to single virus detection.

The urethra is more narrow in younger patients, which is closely associated with the CBI failure of HC. Lucila et al. [11]. reviewed 133 recipients undergoing HSCT and found that younger patients were more likely to develop BK polyomavirus related HC in multivariate regression analysis and had a worse prognosis. Nevertheless, in Johnston,s study, age as a continuous variable was not associated with HC treatment outcome (*P* = 0.0773) and HC grade (*P* = 0.0721) [1]. In this study, multiple regression analysis was carried out in the multi-age group of HSCT. It was found that the age of fewer than 14.5 years old was an independent risk factor for the failure of CBI in HC patients.

For the applicability of the research results, the study excluded grade I HC patients, for these patients had no gross hematuria or CBI. The limitations of this study are as follows: 1. This study is retrospective and all data was collected from a single-center. However, our center has received a large number of HSCT patients from all over the country. The source of HSCT patients is rather sufficient. Clinically factors can still be collected from this single-center, which may guide the current treatment and future investigation. 2. The results of CRP, serum albumin, and so on are single time point values, which may vary with time point. But this study chose the initial gross hematuria time stage point as the time point to describe the study for the best fit for the clinical pathway. Despite these limitations, this study revealed the effect of serum CRP on the failure of CBI in HC patients.

## Conclusions

HC, as one of the complications of HSCT, is not rare. Compared with radiation cystitis, HSCT related HC has unique clinical characteristics. The study identified CRP > 8.89 ng/ml, age < 14.5 years, and onset time of HC after HSCT > 37d are independent factors for failure of CBI, which could be combined to allow stratification of HC after HSCT patients into low-, intermediate- and high-risk subgroups of CBI failure. In the clinical pathway, we can avoid the dilemma of surgical intervention by taking more targeted and active interventions. In the future, we need to expand the sample size and conduct the prospective, multi-center study to gradually improve the predicted model.

## Data Availability

The datasets used and/or analysed during the current study are available from the corresponding author on reasonable request.

## Abbreviations

CBI: Continuous bladder irrigation
CRP: C-reactive protein
CY: Cyclophosphamide
CMV: Cytomegalovirus
GVHD: Graft-versus-host disease
HSCT: Hematopoietic stem cell transplantation
HC: Hemorrhagic cystitis
ROC: Receiver operating characteristic
